# Fully Printed High-Performance n-Type Metal Oxide Thin-Film Transistors Utilizing Coffee-Ring Effect

**DOI:** 10.1007/s40820-021-00694-4

**Published:** 2021-08-03

**Authors:** Kun Liang, Dingwei Li, Huihui Ren, Momo Zhao, Hong Wang, Mengfan Ding, Guangwei Xu, Xiaolong Zhao, Shibing Long, Siyuan Zhu, Pei Sheng, Wenbin Li, Xiao Lin, Bowen Zhu

**Affiliations:** 1 Key Laboratory of 3D Micro/Nano Fabrication and Characterization of Zhejiang Province, School of Engineering, Westlake University, Hangzhou, 310024 China; 2 Zhejiang University, Hangzhou, 310027 China; 3 Key Laboratory of Wide Band Gap Semiconductor Technology, School of Microelectronics, Xidian University, Xian, 710071 China; 4 School of Microelectronics, University of Science and Technology of China, Hefei, 230026 China; 5 Instrumentation and Service Center for Physical Sciences, Westlake University, Hangzhou, 310024 China; 6 Institute of Advanced Technology, Westlake Institute for Advanced Study, Hangzhou, 310024 China; 7 School of Science, Westlake University, Hangzhou, 310024 China

**Keywords:** Printed electronics, Indium tin oxide, Thin-film transistors, Coffee-ring effect, NMOS inverters

## Abstract

Metal oxide thin-films transistors (TFTs) produced from solution-based printing techniques can lead to large-area electronics with low cost. However, the performance of current printed devices is inferior to those from vacuum-based methods due to poor film uniformity induced by the “coffee-ring” effect. Here, we report a novel approach to print high-performance indium tin oxide (ITO)-based TFTs and logic inverters by taking advantage of such notorious effect. ITO has high electrical conductivity and is generally used as an electrode material. However, by reducing the film thickness down to nanometers scale, the carrier concentration of ITO can be effectively reduced to enable new applications as active channels in transistors. The ultrathin (~10-nm-thick) ITO film in the center of the coffee-ring worked as semiconducting channels, while the thick ITO ridges (>18-nm-thick) served as the contact electrodes. The fully inkjet-printed ITO TFTs exhibited a high saturation mobility of 34.9 cm^2^ V^−1^ s^−1^ and a low subthreshold swing of 105 mV dec^−1^. In addition, the devices exhibited excellent electrical stability under positive bias illumination stress (PBIS, Δ*V*_th_ = 0.31 V) and negative bias illuminaiton stress (NBIS, Δ*V*_th_ = −0.29 V) after 10,000 s voltage bias tests. More remarkably, fully printed n-type metal–oxide–semiconductor (NMOS) inverter based on ITO TFTs exhibited an extremely high gain of 181 at a low-supply voltage of 3 V, promising for advanced electronics applications.

**Supplementary Information:**

The online version contains supplementary material available at 10.1007/s40820-021-00694-4.

## Highlights

- Fully inkjet-printed transparent high-performance thin-film transistors (TFTs) with ultrathin indium tin oxide (ITO) as semiconducting channels were achieved.
- The energy band alignment at ITO/Al_2_O_3_ channel/dielectric interface was investigated by in-depth spectroscopy analysis.
- Fully printed n-type metal–oxide–semiconductor inverters based on ITO TFTs exhibited extremely high gain of 181 at a low-supply voltage of 3 V, promising for applications in advanced electronic devices and circuits.

**Supplementary Information:**

The online version contains supplementary material available at 10.1007/s40820-021-00694-4.

## Graphical abstract

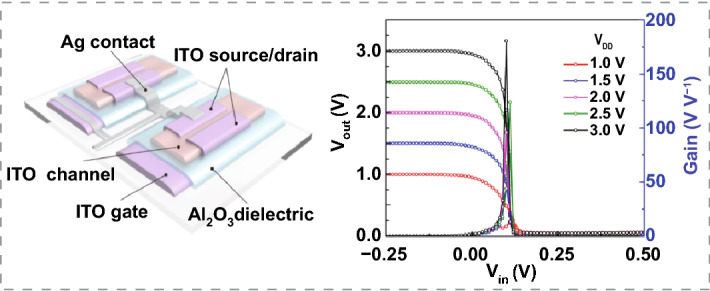

## Introduction

Metal oxide (MO) thin-film transistors (TFTs) have emerged as core components for large-area electronics including transparent displays, optoelectronics, and electronic skins [1–3]. Compared with their counterparts such as amorphous silicon and organic semiconductors, metal oxide semiconductors (MOS) exhibit some intriguing properties, such as high carrier mobility, wide bandgaps, and high optical transparency [4–7]. In addition, MOSs are more compatible with low-cost vacuum-free manufacturing techniques as they can achieve high device performance under relatively low processing temperature (from room temperature to 350 °C) [8–11].

To this end, solution-processed MO TFTs have been extensively studied [11–15]. The typical solution-processing technologies include screen printing, spraying coating, spin coating, and inkjet printing. In particular, inkjet printing offers advantages including direct alignment, multilayer maskless patterning, high efficiency in materials choices, fast and noncontact processing, all of which are desirable for the manufacturing of large-area low-cost electronics [16–20].

In the typical inkjet printing process, when an ink droplet dries on the substrate, the solutes tend to deposit along the periphery, resulting in concave films with thin center and thick edges. Such phenomenon is known as the “coffee-ring effect” and is conventionally unwelcoming because it not only affects film uniformity but deteriorates electrical performance [21–23]. Originated from surface tension, convective flow, and environmental conditions, coffee-ring effect is nearly inevitable in inkjet printing processes [24, 25]. To date, extensive efforts have been devoted to suppressing coffee-ring formation to achieve better film uniformity, which however, turns out to be with little success. The mobilities of most fully printed MO TFTs are relatively low (0.04–12.9 cm^2^ V^−1^ s^−1^) [26–29], limiting their applications in displays and circuits.

**Fig. 1:**
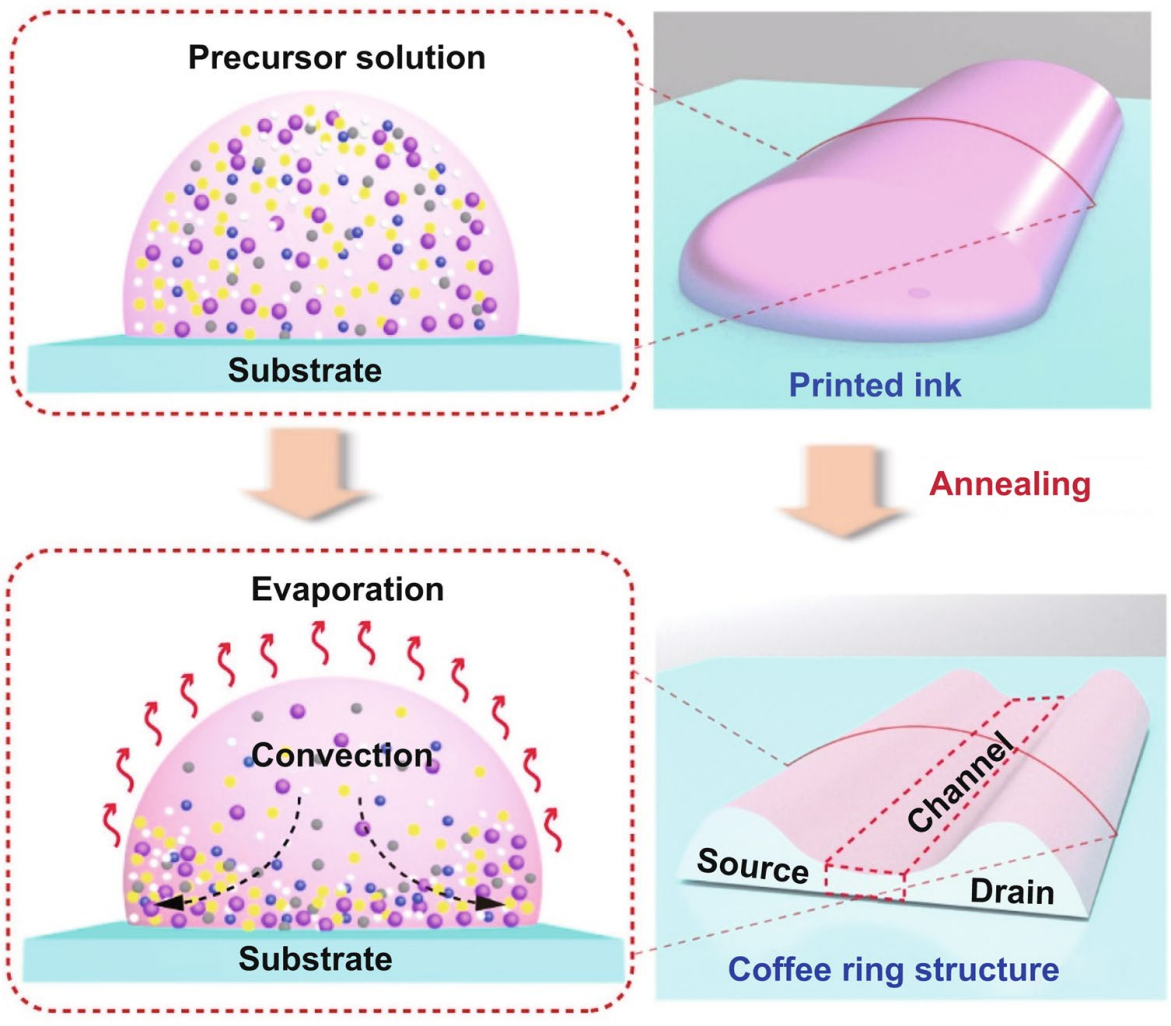
Schematic illustration showing the formation process of the coffee-ring structure. During annealing process, the solute moved to the edges due to capillary flow, forming coffee-ring patterns

Here, on the contrary, we propose a novel printing approach that can take full advantage of such notorious “coffee-ring” effect to achieve high-performance indium tin oxide (ITO)-based TFTs and logic inverters. ITO has recently risen an excellent active channel material with high-performance mobility, beyond its conventional role as transparent conducting electrodes [30–34]. As a typical “coffee-ring” structure is featured with thin film in the center and thick ridges at the edge, in our approach, we directly integrated ITO TFTs from the coffee-ring structure as-printed (Fig. 1). The ultrathin ITO film (~10 nm in thickness) in the ring center can work as excellent semiconducting channel [31–37], while the thick ITO ridges can serve as parts of source/drain (S/D) electrodes. Benefited from the ultrathin nature of the ITO channels and the integrated design, our fully inject-printed ITO TFTs demonstrated a high optical transparency (~90%) and outstanding electrical property with a high saturation mobility (*µ*_sat_) of 34.9 cm^2^ V^−1^ s^−1^, a low subthreshold swing (*SS*) of 105 mV dec^−1^, a near-zero turn-on voltage (*V*_on_) of −0.09 V, and a good current on/off ratio (*I*_on/off_) of ~10^5^. More impressively, the fully inkjet-printed NMOS inverter based on two ITO TFTs exhibited an extremely high gain of 181 at a low-supply voltage of 3 V, outperforming other documented solution-processed inverters and is even comparative to short-channel devices fabricated via vacuum-based lithography processes. Combining the merits of additive manufacturing, capability of direct patterning, and outstanding electrical performance, our fully inkjet-printed ITO TFTs hold great promise for future advanced thin-film electronics.

## Experimental Section

### Preparation of Precursor Inks and Printing Techniques

Precursor inks were prepared by dissolving corresponding metal salts in a mixed solvent of 2-methoxyethanol (2-ME), and ethylene glycol (EG) with a volume ratio of 1:1. EG was added to improve the viscosity of the inks, an important parameter to regulate the diffusion features on substrates, thus facilitating the printing processes. In addition, to optimize the electrical performance of solution-processed metal oxide films, we introduced two additives, acetylacetone (AcAc) and ammonium hydroxide (NH_4_OH), into the metal oxide precursor inks to boost the exothermic combustion reactions. In a typical printing process, indium tin oxide (ITO) precursor ink was prepared by dissolving In(NO_3_)_3_·xH_2_O (99.999%, Sigma-Aldrich) and SnCl_2_·2H_2_O (99.99%, Sigma-Aldrich) into a mixture of 2-methoxyethanol (2-ME, Alfa Aesar, 99.3%) and ethylene glycol (Alfa Aesar, 99%) with a volume ratio of 1:1. The molar ratio of In to Sn was kept at 9:1 in precursors with different concentrations (0.2 M for channel and 0.5 M for contacts). The Al_2_O_3_ precusor solution was prepared by dissolving aluminum nitrate nonahydrate (Al(NO_3_)_3_·9H_2_O, Sigma-Aldrich, 99.997%) in mixed solvent of 2-ME and ethylene glycol (1:1, v:v). Then, 0.2 m acetylacetone (AcAc, Alfa Aesar, 99%) and 0.1 M ammonium hydroxide (NH_4_OH, 28.0% NH_3_ in water, Alfa Aesar) were added in precursor inks solutions as additives to facilitate combustion reactions. Both ITO and Al_2_O_3_ precursor inks were stirred at room temperature for 12 h and filtered through nylon syringe filter (0.22 μm pore size) before use.

### Device Fabrication and Characterization

A Dimatix (DMP-2850) printer was used to print different films with desired patterns. During printing, the substrate temperature of the printer was set at 30 °C. ITO TFTs were printed with bottom-gate, top-contact configuration on glass substrates. The glasses were cleaned by sequential ultrasonication in acetone, isopropanol, and deionized (DI) water for 10 min before use, respectively. Subsequently, the glasses were treated with oxygen plasma for 5 min. Prior to inkjet printing of the first layer of the ITO gate electrode, a thin buffer layer of Al_2_O_3_ was spin-coated on substrates at 3000 r min^−1^ for 30 s and then annealed at 350 ℃ in air for 1 h, to better control the shape of the inks. In a typical printing process, 0.5 M ITO ink was firstly printed as a gate electrode with a linewidth of ~150 μm, and subsequently sintered at 350 °C for 1 h. Subsequently, the gate dielectric was deposited by printing 0.6 M Al_2_O_3_ precursor inks onto the ITO gate contacts and baked at 200 °C for 10 min; the process was repeated three times to build up desired thickness and sufficient insulating property, and then the films were annealed at 350 °C for 1 h. Next, the ultrathin (~10 nm) ITO channel layers were printed with 0.2 M ink, above the ITO gate contacts, onto the gate dielectric layers, followed by annealing at 350 °C for 1 h. Finally, 0.5 M ITO precursor ink was printed along the ridge edges of ITO channel layers and subsequently annealed 350 °C for 1 h. All the annealing processes were conducted on a hot plate in air. After the annealing, the last printed ITO films merged with the ridge areas of preceding ITO channel layers, forming final paralleling S/D contacts. The channel width (W) and length (L) of the resulting TFTs were about 600 and 40 μm, respectively. Commercially available silver inks (Silverjet DGP 45HTG, ANP Co., LTD) were used to connect two ITO TFTs in fabricating NMOS inverters. The silver inks were annealed at 100 ℃ for 1 h in air after printing.

The electrical properties of the printed devices were carried out using semiconductor parameter analyzer (Keithley 4200 SCS) and/or source meter (Agilent B2912A) integrated with probe station system in ambient atmosphere in dark at room temperature. The NBIS and PIBS were performed in air at room temperature under white LED light illumination (3000 lx) with the applied gate bias of −1 and +1 V, respectively.

The values of saturation mobility (*µ*_sat_) and subthreshold swing (SS) were extracted from the transfer characteristics using the gradual channel approximation model as following:

1 $$\mu_{{{\text{sat}}}} = \frac{2L}{{{\text{WC}}_{{{\text{ox}}}} }}\left( {\frac{{\partial \sqrt {I_{{{\text{ds}}}} } }}{{\partial V_{{{\text{gs}}}} }}} \right)^{2}$$

2 $${\text{SS}} = \frac{{\partial V_{{{\text{gs}}}} }}{{\partial \left( {\log_{10} I_{{{\text{ds}}}} } \right)}}$$

where *I*_ds_ denotes the drain current, *C*_ox_ is the capacitance of the gate dielectric, *W* and *L* are the channel width and length, and *V*_gs_ denotes the gate voltage. Based on the above SS value, the interfacial trap density (*D*_it_) between the semiconductor and the gate dielectric was calculated using the following equation:

3 $$D_{{{\text{it}}}} = \left( {\frac{{{\text{SS}}\log_{10} e}}{{{{kT} \mathord{\left/ {\vphantom {{kT} q}} \right. \kern-\nulldelimiterspace} q}}} - 1} \right)\frac{{C_{{{\text{ox}}}} }}{q}$$

where *q* and *T* are the electron charge and measurement temperature (300 K), *k* is Boltzmann constant.

### Materials Characterization

Surface topography of printed films was measured via ac-mode atomic force microscopy (AFM, Cipher ES, Oxford Instruments) and surface profilometer (P-7, KLA-Tencor). AFM samples for height profile were prepared by photolithography and wet etching with diluted hydrochloric acid (1:10 in water, v:v). The chemical structure of the ITO was examined by X-ray photoelectron spectroscopy (XPS, ESCALAB XI^+^, Thermo Fisher Scientific) using a monochromatic Al K_α_ X-ray source. XPS peaks were calibrated by taking C 1s reference at 284.6 eV. XPS depth profile analysis was performed by mild, destructive in-situ sputter etching using a 2000 eV defocused Ar^+^ beam, and monatomic mode to achieve the required depth resolution. Transmission electron microscopy (TEM, Titan Themis 200, FEI) equipped with an energy dispersive X-ray spectrometer (EDS, super-X, Bruker,) was used to obtain the structure and chemical information on the printed oxide TFT. The crystallization and structural information of the films were obtained using X-ray diffraction (XRD, D8 Advance, Bruker) with Cu K_α_ radiation. The optical transmittance of the printed TFTs were determined with a UV–Vis spectrophotometer (UV-3600Plus, Shimadzu) with spectrum ranging from 300 to 1200 nm.

## Results and Discussion

### Printing Technique and Transistor Performance

**Fig. 2:**
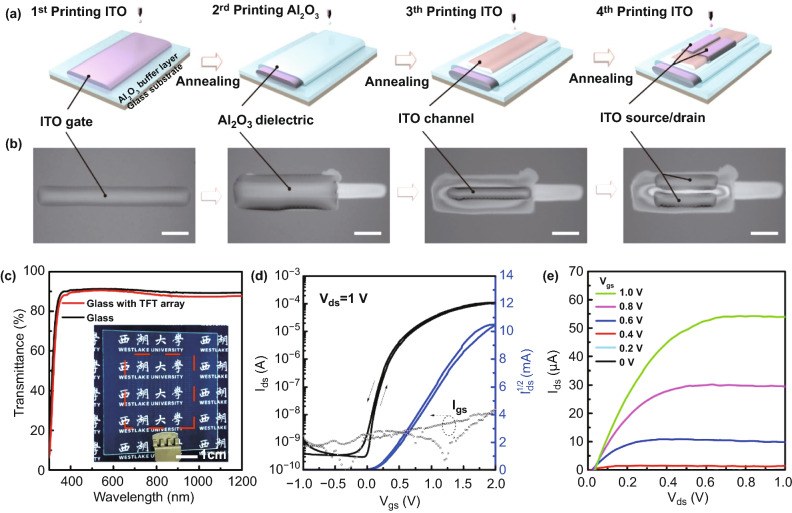
Process flow of fully inkjet-printed ITO TFTs and electrical characteristics. **a** Schematic illustration of the printing processes. Each film was annealed at 350 °C for 1 h after printing. **b** Corresponding optical images of each film right after printing and before annealing. Scale bars: 200 µm. **c** Optical transmission spectrum of fully printed TFT array on glass. Inset shows a digital photo of a glass with ITO TFT arrays. The red dashes indicate the distribution area of the printed TFT devices. **d** Transfer curves of the fully-printed TFT with ITO channel thickness of ~ 10 nm, and channel length/width = 40/600 µm. **e** Output characteristics of the ITO TFT with *V*_gs_ changed from 0 to 2 V in steps of 0.5 V, showing clear pinch-off behaviors

The ITO TFTs were printed with bottom-gate, top-contact configuration on glass substrates, with ITO as both channel and contact electrodes, and printed Al_2_O_3_ as gate dielectric, as illustrated in Fig. 2a and detailed in Supporting Information. The whole printing process was conducted in ambient environment, and the corresponding optical images of each layer right after printing are presented in Fig. 2b. As shown in Fig. S1, the printed ITO film exhibited a typical coffee-ring structure, where the middle “valley bottom” area was thinner (~10 nm in thickness), and the edges were relatively thicker (~15 nm).

The fully inkjet-printed TFT array on glass showed high optical transparency approaching 90% in the visible spectrum, as depicted in the photo and the ultraviolet–visible (UV–Vis) transmittance spectra (Fig. 2c). The dielectric properties of printed Al_2_O_3_ film are shown in Fig. S2. Figure 2d, e shows the typical transfer and output characteristics of the fully inkjet-printed ITO transistors. The ITO TFT exhibited superior electrical performance of a *µ*_sat_ of 34.9 cm^2^ V^−1^ s^−1^, a *V*_on_ of −0.09 V, a low *SS* of 105 mV dec^−1^, a high *I*_on/off_ of 10^5^, low-operation voltage range of <2 V, and negligible hysteresis. To evaluate the device uniformity of fully-printed devices, the statistics of *µ*_sat_, threshold voltage (*V*_th_), *SS*, and *I*_on/off_ of different ITO TFTs were presented (Fig. S3). These devices exhibited a mobility of 33.1 ± 2.4 cm^2^ V^−1^ s^−1^ with the highest mobility of 36.0 cm^2^ V^−1^ s^−1^, a *V*_th_ of 0.06 ± 0.03 V, low *SS* of 110 ± 20 mV dec^−1^, and *I*_on/off_ > 10^4^. In addition, to further study the printed devices uniformity from batch to batch and across different locations, we tested 40 devices from 2 batches at diffferent locations. The statistical results of the *μ*_sat_ and *V*_th_ values are shown in Fig. S4, which exhibit high uniformity with deviations <15%, indicating outstanding uniformity and reliability of the printing methods. The outstanding performance of the fully printed ITO TFTs indicated low density of impurity and defect states at the channel/dielectric interface, and the extracted interface trap density (*D*_it_) of semiconductor and gate dielectric is only 6.5 × 10^11^ cm^−2^ eV^−1^. In comparison, ITO TFTs fabricated on Si/SiO_2_ substrates showed *μ*_sat_ = 11.8 cm^2^ V^−1^ s^−1^ with higher trap density (*D*_it_ = 5.3 × 10^12^ cm^−2^ eV^−1^) (Fig. S5).

To characterize the electrical reliability of fully printed ITO TFTs, the negative/positive bias stress (NBS/PBS) and negative/positive bias stress illumination (NBIS/PBIS) tests were performed in air without device passivation or encapsulation. The ITO TFTs were subjected to voltage bias of *V*_gs_ =  ±3 V at room temperature. As depicted in Fig. S6, the *V*_th_ of ITO device only exhibited minor shifts with −0.17 V at NBS and 0.24 V at PBS under stress time of 4000 s, respectively. For NBIS/PBIS, the devices were tested in ambient conditions under white light illumination (3000 lx) for a duration of 10,000 s, and a Δ*V*_th_ of −0.29 V (NBIS) and 0.31 V (PBIS) was observed (Fig. S7). The small Δ*V*_th_ shift of ITO TFTs indicates robust NBIS and PBIS stability.

### Thin-Film Materials and Interface Analysis

**Fig. 3:**
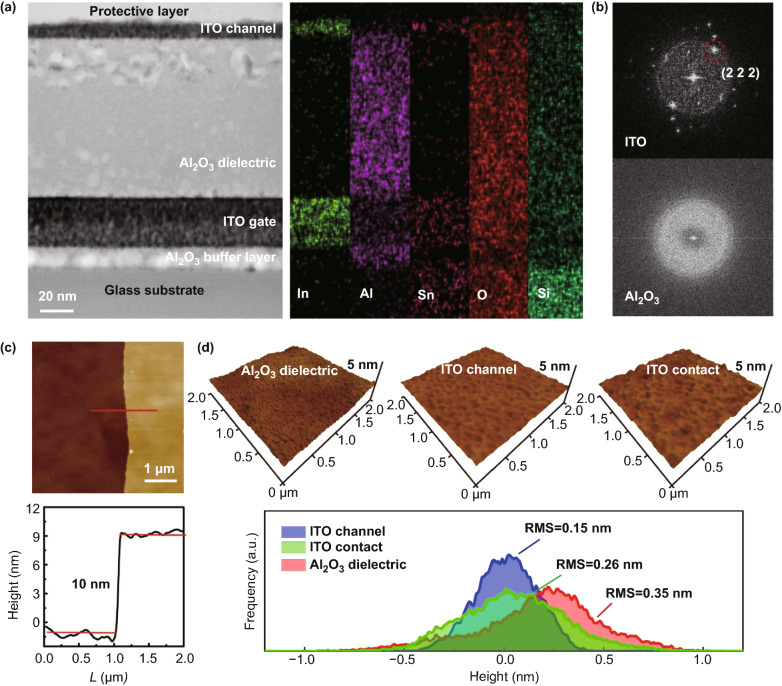
Material characterization of inkjet-printed metal oxide films. **a** Cross-sectional TEM image (left) and corresponding EDS mapping (right) of elements indium (In), aluminum (Al), tin (Sn), oxygen (O), and silicon (Si). **b** FFT patterns obtained from the selected areas of the ITO and Al_2_O_3_ layers. ITO exhibits nanocrystalline and Al_2_O_3_ holds amorphous structures, and the lattice spacing of 0.29 nm corresponds to the (2 2 2) crystal plane of ITO. **c** AFM image and height profile of printed ITO channel film, showing an ultrathin thickness of ~ 10 nm. **d** AFM images showing the surface morphology (top) and height distributions (bottom) of Al_2_O_3_ dielectric, ITO channel, and ITO contacts. The RMS values of ITO contact and Al_2_O_3_ films are 0.26 and 0.35 nm, respectively. And ITO channel exhibits a narrower height distribution with RMS of only 0.15 nm

To investigate the origin of high-performance ITO devices, we used transmission electron microscope (TEM) to characterize the structure of fully printed ITO TFTs. The cross-sectional TEM image is presented in Fig. 3a, clearly showing the layered structures of Al_2_O_3_ buffer layer, ITO gate, Al_2_O_3_ gate dielectric, and ITO channel layers (from bottom to top). The ITO channel layer showed an ultrathin thickness of ~10 nm. The corresponding high-resolution energy-dispersed X-ray spectra (EDS) further confirmed the material composition and the multilayer structures of the oxide films (Fig. 3a, right). The fast Fourier transform (FFT) pattern revealed that ITO films were composed of nanocrystals with lattice spacing of 0.29 nm, corresponding to the (222) crystal plane of ITO (Fig. 3b, top), while the Al_2_O_3_ film was amorphous (Fig. 3b, bottom). The polycrystalline and amorphous properties of ITO and Al_2_O_3_ films were also confirmed by X-ray diffraction (XRD) analysis (Fig. S8). The amorphous nature of Al_2_O_3_ film is beneficial for improving insulating properties and suppressing the leakage current, because the grain boundaries in polycrystalline structures provide channels for leakage current [27]. The film thickness (~10 nm) of printed ITO active channel was also confirmed by atomic force microscopy (AFM), as shown in Fig. 3c. Surface roughness is another important parameter indicating the quality of printed films. We examined the surface morphology of printed oxide films with AFM (Fig. 3d), and all the films exhibited highly flat surfaces. The root-mean-square (RMS) roughness values were only 0.35, 0.15, and 0.26 nm over 2 × 2 µm^2^ area for Al_2_O_3_ dielectric, ITO channel, ITO contact films, respectively. These smooth surfaces contributed to the high mobility and stability of ITO TFTs by suppressing interface scattering and charge trapping [32].

**Fig. 4:**
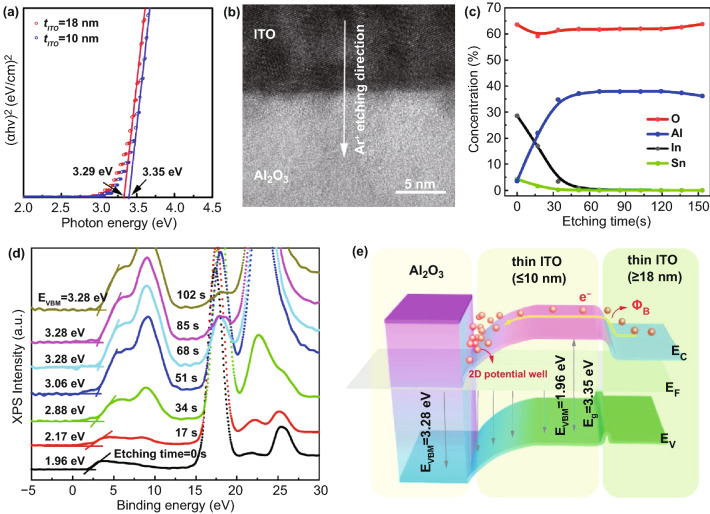
Energy band analysis of ITO/Al_2_O_3_ interfaces. **a** Tauc plots of ITO films with different thicknesses of 10 and 18 nm. **b** Cross-sectional TEM image of ITO/Al_2_O_3_ interface. The arrow indicates the Ar etching direction (from top ITO to bottom Al_2_O_3_). **c** Elemental concentration of In, Sn, Al, and O in the ITO/Al_2_O_3_ films as a function of etching time, extracted from the XPS depth profiles. **d** Depth-resolved VBM spectra based on in-situ XPS measurements with different Ar etching time. The VBM energy increased gradually from 1.96 eV of ITO surface and became stable at 3.28 eV of bulk Al_2_O_3_. **e** Energy band diagram of the thick ITO/thin ITO/Al_2_O_3_ heterostructure reconstructed based on UV–Vis and XPS depth spectra. The band bending at the ITO/Al_2_O_3_ interfaces induced a 2D potential well confining free electrons. And a barrier *Φ*_*B*_ exists at ITO channel and electrode layers due to the difference in bandgap

To study the energy band alignment at ITO/Al_2_O_3_ interface, in-depth spectroscopy analysis was performed. The ITO bandgap widened from 3.29 to 3.35 eV with film thickness decreased from 18 to 10 nm (Fig. 4a), which is in good agreement with the theoretical prediction of energy quantization [38, 39]. To further reveal the influence of film thickness on carrier concentration, we performed XPS analysis on both 18- and 10-nm-thick ITO films. Figure S9 shows the O 1*s* XPS spectra of these films. The O 1*s* peak could be deconvoluted into three peaks at 530.1 ± 0.3, 531.6 ± 0.2, and 532.9 ± 0.3 eV, which were assigned to the lattice oxygen atoms (M–O) bonding, oxygen vacancies (*V*_*O*_), and oxygen atoms in hydroxyl groups (M–OH), respectively [40]. Oxygen vacancies were produced by defect states that acted as donor-like states, which are the major source of free carriers in metal oxide semiconductors [41]. The ratios of *V*_*O*_ were 30.5% and 42.3% for 10- and 18-nm-thick ITO films, respectively. Thicker ITO film possessed more *V*_*O*_ because the front channel is less likely exposed to the ambient oxygen during annealing, resulting in anoxic states [42]. The large proportion of *V*_*O*_ results in a higher carrier concentration, leading to reduced resistivity of the ITO films. The transfer characteristic of TFTs with different ITO channel thickness also confirmed such a transition of ITO from semiconducting to metallic behaviors (Fig. S10). As the channel thickness increases, the *μ*_sat_ increases, and *V*_th_ shifts negatively, and the 18-nm-thick channel-based device showed very poor switching behavior. In addition, contact resistance can also affect the electrical performance of TFT. The barrier height between the channel and the source/drain electrode could strongly influence the contact resistance, and the extracted contact resistance of 18-nm-thick ITO is much lower than that of 10-nm-thick ITO by gated four-probe (GFP) method (Fig. S11). Also, the increase of gate voltage could result in the decrease of contact resistance [43].

To unveil the chemical composition and local chemical binding states of ITO/Al_2_O_3_ heterostructure, in-situ XPS analysis with mild Ar etching was performed to obtain depth profiles. A cross-sectional TEM image showing the interface and etching direction is illustrated in Fig. 4b. As shown in Fig. 4c, the concentration of In and Sn reduced gradually (~0% after etching for 68 s), while that of Al increased instead, indicating the top ITO films were successfully etched. The corresponding XPS depth spectra of In-3*d*, Sn-3*d*, Al-2*p*, and O-1*s* with respect to varied etching time are shown in Fig. S12. Figure 4d depicts the depth-resolved valence band maximum (VBM) spectra based on the in-situ XPS analysis, where distinct spectral differences can be observed. The VBM energies increased gradually from surface ITO (1.96 eV) to bulk Al_2_O_3_ region (3.28 eV) with increased Ar etching time from 0 to 68 s, indicating the variation in the binding states of In approaching the ITO/Al_2_O_3_ interface [44]. With etching time beyond 68 s, the VBM energies became stable at 3.28 eV, corresponding to the bulk Al_2_O_3_ region. The all-energy band arrangement of ITO/Al_2_O_3_ heterostructure was illustrated in Fig. 4e. The interfacial downward band bending created a two-dimensional (2D) potential well that draws and confines free electrons supplied from the interface. This observation agrees with previous studies on heterostructures of In_2_O_3_/Al_2_O_3_ [44] and In_2_O_3_/ZnO [38, 39]. In addition, because of the bandgap difference between thick ITO electrode and thin ITO channel layers, a barrier *Φ*_*B*_ will form at the electrode/channel interface (Fig. 4e), which can suppress off current and influence threshold voltage of devices [42].

### Fully Printed High-Gain NMOS Inverter

**Fig. 5:**
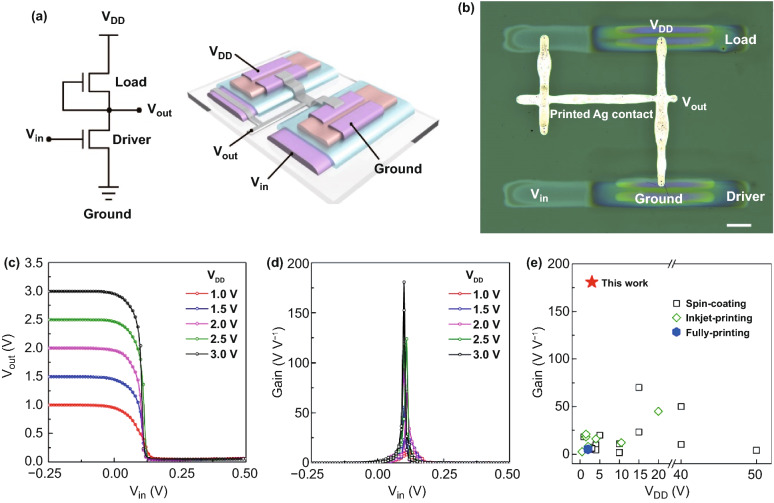
Fully inkjet-printed NMOS logic inverter based on ITO TFTs. **a** Circuit diagram and schematic illustration of the NMOS inverter structure. **b** An optical image of logic inverter based on two ITO TFTs connected by inkjet-printed silver contacts. Scale bar: 200 µm. **c** Input–output (*V*_in_*–V*_out_) voltage characteristics of the inverter under various supply voltages. **d** Corresponding gains of the NMOS inverter, showing a maximum gain of 181 at *V*_DD_ = 3 V. **e** Benchmark of inverter performance as a function of supply voltage for inverters based on metal oxide semiconductor TFTs with different solution-processing techniques and the fully inkjet-printed ITO TFTs in this work. The data were taken from Table S1

To explore the potential applications of fully printed ITO TFTs, we integrated a NMOS inverter using two ITO transistors printed on glass substrate. The circuit diagram and device structure are illustrated in Fig. 5a. Printed silver electrodes were used to bridge the contacts of two ITO TFTs. An optical image of the inverter is presented in Fig. 5b, the top TFT (load TFT) worked as a depletion load to limit the current flowing through the bottom TFT (driver TFT). Figure 5c shows the voltage transfer characteristics of the printed inverter at different supply voltages. When the input voltage (*V*_in_) was lower than the threshold voltage (logic “0”), the output voltage (*V*_out_) was equal to *V*_DD_ (logic “1”). With *V*_in_ exceeding the threshold voltage (logic “1”), the *V*_out_ decreased abruptly to ~0 V (logic “0”), indicating the driver TFT was in a nearly short-circuit state due to the high mobility and large on-state current of ITO TFT. This implies that the load and driver TFTs showed excellent on-state and off-state behaviors in the inverter. Remarkably, the NMOS inverter exhibited the maximum transfer gain of 181 at a low-supply voltage of *V*_DD_ = 3 V, and still a high gain of 96 at a lower *V*_DD_ = 2 V (Fig. 5d), by virtue of the high mobility, small SS, and low off-state current of the fully printed ITO TFTs. Our inverter outperformed other solution-processed metal oxide TFTs reported previously (Fig. 5e). A detailed comparison among inverters based on different solution-processing techniques is presented in Table S1. The fully inkjet-printed high-gain inverter opens new opportunities for boosting inkjet printing techniques in delivering high-performance low-power circuits.

## Conclusions

In conclusion, we report fully inkjet-printed high-performance electronics by integrating the coffee-ring structured ITO generated during the printing process. In such ITO TFTs, the ultrathin ITO film (~10 nm in thickness) in the center showed widened bandgap and reduced oxygen vacancies, which could serve as excellent semiconducting channels. The thick ITO ridges, on the other hand, served as the parts of source/drain (S/D) electrodes. The fully printed ITO TFTs exhibited high transparency (90% in visible spectrum), low-operation voltage (<3 V), high mobility (34.9 cm^2^ V^−1^ s^−1^), and low subthreshold swing (105 mV dec-1). The fully printed ITO TFTs exhibited outstanding electrical stability with small Δ*V*_th_ values of −0.17, 0.24, −0.29, and 0.31 V for NBS, PBS, NBIS, and PBIS tests, respectively. Also, the printed devices exhibited outstanding uniformity and reliability. More impressively, our fully printed NMOS inverter exhibited an extremely high gain (181) at a low-supply voltage (V_DD_ = 3 V), outperforming other solution-processed metal oxide counterparts, and was comparative to the devices fabricated from vacuum processes. Our work bridged the gap between high-performance TFTs and the solution-based printing technologies, holding great potential for the further application in large-area, low-cost electronic devices and circuits.

## Supplementary Information

Below is the link to the electronic supplementary material.

Supplementary file1 (PDF 1280 kb)
