# Supplementary material for: Fully Printed High-Performance n-Type Metal Oxide Thin-Film Transistors Utilizing Coffee-Ring Effect
Source: Nanomicro Lett. 2021 Aug 3;13:164. doi: 10.1007/s40820-021-00694-4 (PMC8333237; doi:10.1007/s40820-021-00694-4)
Supplement: Supplementary file 1 — Supplementary file1 (PDF 1280 kb) [file 40820_2021_694_MOESM1_ESM.pdf]

Supporting Information for

Fully Printed High-Performance n-Type Metal Oxide Thin-Film Transistors Utilizing Coffee-Ring Effect

Kun Liang[1, 2], Dingwei Li[1, 2], Huihui Ren[1, 2], Momo Zhao[1, 3], Hong Wang[3], Mengfan Ding[4], Guangwei Xu[4], Xiaolong Zhao[4], Shibing Long[4], Siyuan Zhu[5], Pei Sheng[5], Wenbin Li[1, 6], Xiao Lin[7], and Bowen Zhu[1, 6, *]

[1]Key Laboratory of 3D Micro/Nano Fabrication and Characterization of Zhejiang Province, School of Engineering, Westlake University, Hangzhou 310024, P. R. China

[2]Zhejiang University, Hangzhou 310027, P. R. China

[3]Key Laboratory of Wide Band Gap Semiconductor Technology, School of Microelectronics, Xidian University, Xi'an 710071, P. R. China

[4]School of Microelectronics, University of Science and Technology of China, Hefei 230026, P. R. China

[5]Instrumentation and Service Center for Physical Sciences, Westlake University, Hangzhou 310024, P. R. China

[6]Institute of Advanced Technology, Westlake Institute for Advanced Study, Hangzhou 310024, P. R. China

[7]School of Science, Westlake University, Hangzhou 310024, P. R. China

*Corresponding author. E-mail: zhubowen@westlake.edu.cn (Bowen Zhu)

Supplementary Tables and Figures

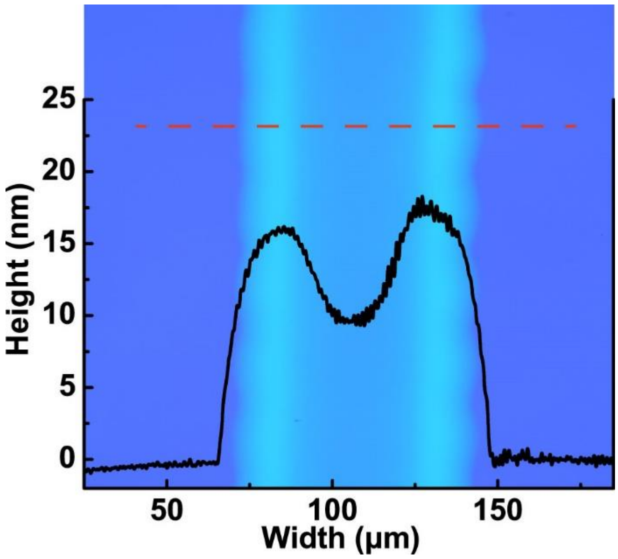

Fig. S1 Optical image and height profile of printed ITO channel. The film was printed with 0.2 M ITO precursor ink, exhibiting typical coffee-ring effect where the center area is thinner than the edges.

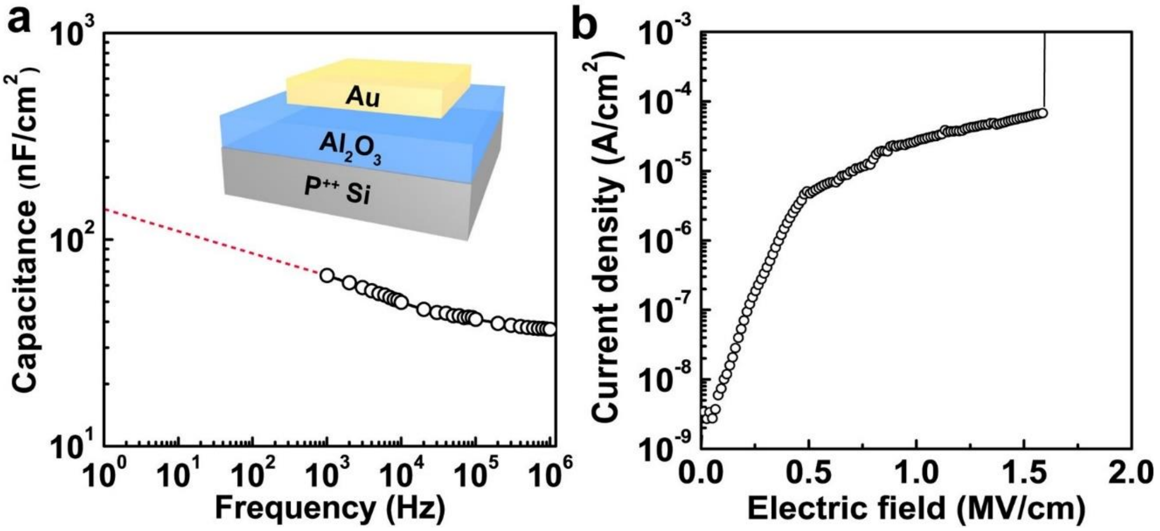

Fig. S2 Electrical properties of printed Al_2O_3 insulating layer based on a metal-insulator-metal (MIM) structure. (**a**) Areal capacitance of Al_2O_3 film in the frequency range from 1 kHz to 1 MHz. The frequency-dependent capacitance behaviors may result from orientation polarization and space charge polarization in solution-processed Al_2O_3 dielectric films. The capacitance of the printed Al_2O_3 films was extrapolated to be 128 nF cm^{-2} at 1 Hz. (**b**) Current density of the printed Al_2O_3 dielectric with applied electric field. The tests were conducted based on a metal-insulator-metal structure with highly doped Si (p^{++}) as bottom electrode, printed Al_2O_3 as insulating layer, and thermal-evaporated Au films via shadow masks as patterned top electrodes. The device holds an area of 100 µm × 100 µm. The Al_2O_3 film exhibited a low leakage current density of 2.3×10^{-5} A cm^{-2} at electric field strength of 1 MV/cm, and breakdown occurred until the electric field reached 1.6 MV cm^{-1}.

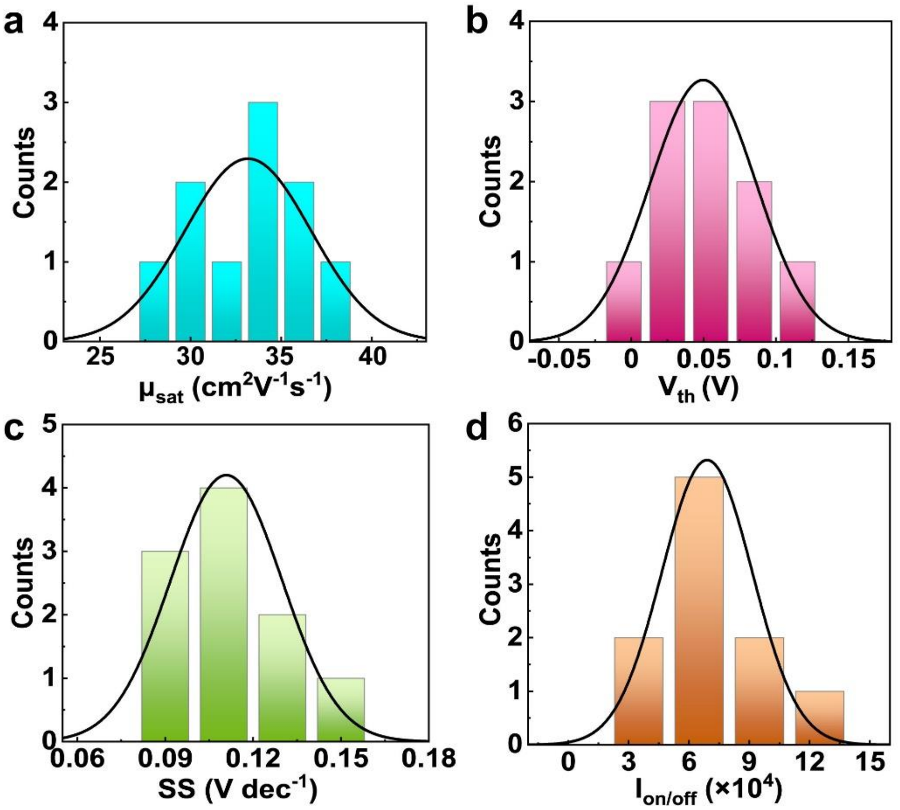

Fig. S3. Histograms showing the distribution of different parameters of the ITO TFTs. (**a**) Distribution of saturation mobility (μ_{sat}). (**b**) Distribution of threshold voltage (V_{th}). (**c**) Distribution of subthreshold swing (SS). (**d**) Distribution of current on/off ratios ($I_{on/off}$)

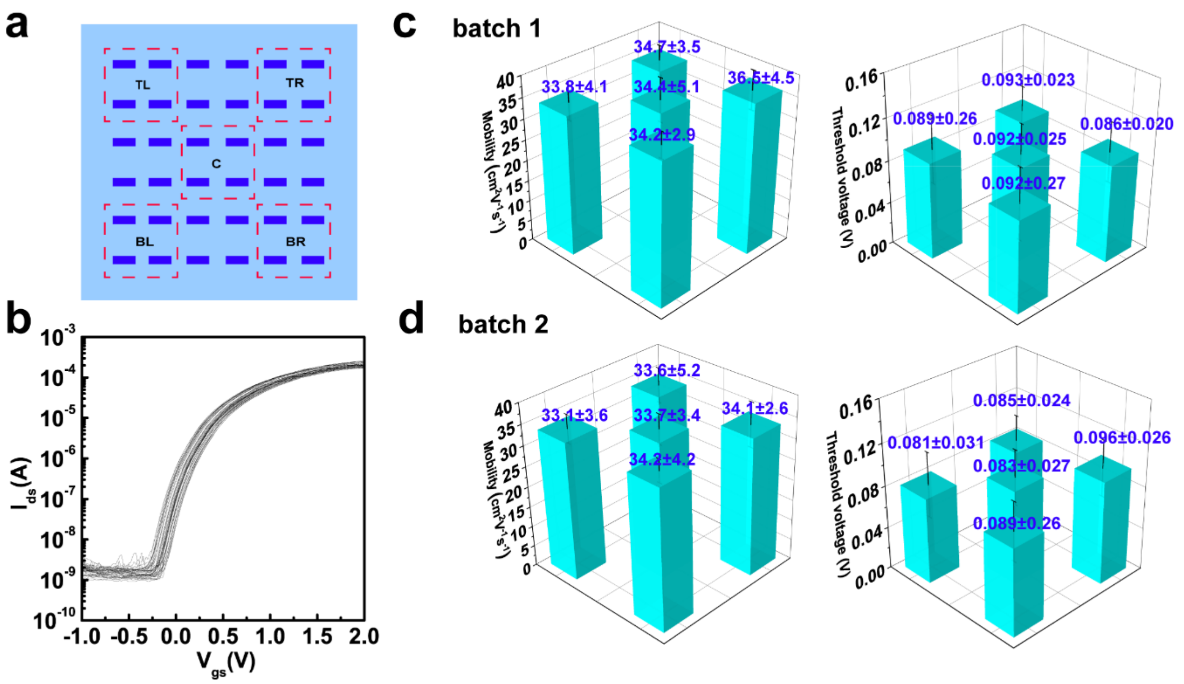

Fig. S4 Probabilistic histograms of the mobility (μ_{sat}) and subthreshold voltage (V_{th}) values of 40 printed devices from 2 batches. (**a**) Devices located at 5 different areas in each substrate were measured: top left (TL), top right (TR), bottom left (BL), bottom right (BR), and center area (C). 4 TFTs at each area were measured. In total, the parameters from 40 TFTs were collected (2 substrates × 5 area × 4 devices). (**b**) Transfer curves for all statistical devices. (**c-d**) Average μ_{sat} and V_{th} values from two batches, showing high uniformity with deviations less than 15%

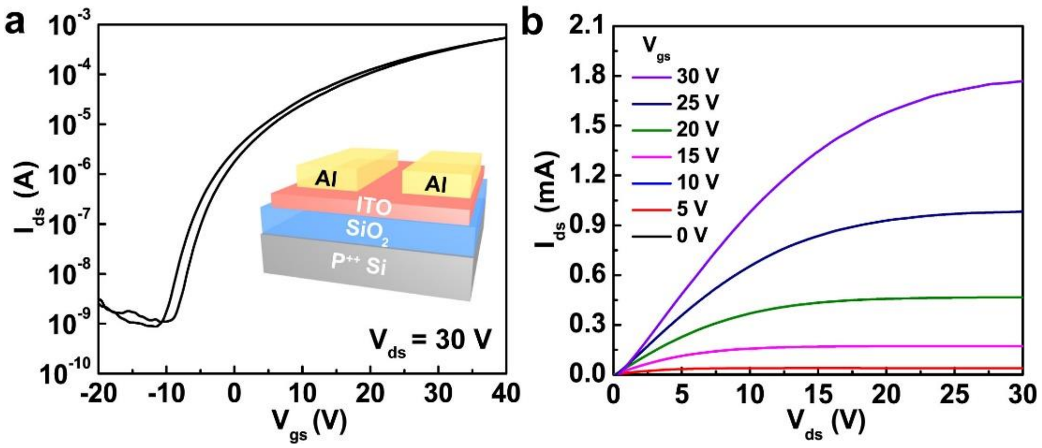

Fig. S5 The electrical characteristics of ITO TFTs on Si/SiO_2 substrates. Transfer (a) and output characteristics (b) of printed ITO TFTs fabricated on Si substrate with 100 nm thick SiO_2 gate dielectric with annealing temperature of 350 ºC in air. The device exhibited electrical properties with μ_{sat} = 11.8 cm^2 V^{-1} s^{-1}, V_{th} = 2.2 V, SS = 1.5 V dec^{-1}, $I_{on/off}$ = 5.2×10^5, and D_{it} = 5.3×10^{12} cm^{-2} eV^{-1}. S/D electrodes of 50 nm thick Al was thermally evaporated via a shadow mask, forming channel L/W = 200/1000 µm.

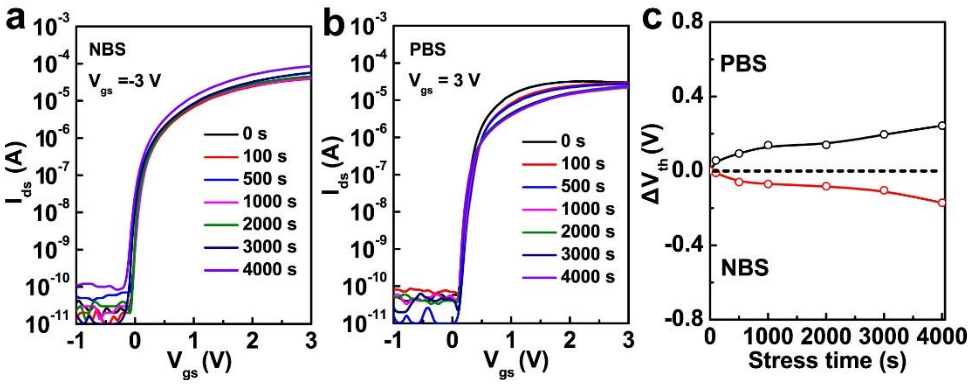

Fig. S6 Electrical stability of fully printed ITO TFTs. (**a, b**) Time-dependent transfer curves of the fully printed ITO TFTs under NBS (V_{gs} = -3 V) (**a**) and PBS (V_{gs} = 3 V) (**b**). (**c**) The detailed plots of threshold voltage shifts (ΔV_{th}) as a function of stress time of the ITO TFT. The negative shift of V_{th} under NBS can be attributed to the attraction of oxygen vacancies at the channel/dielectric interface and the repelling of electrons towards back channel. And the positive shift under PBS results from the electron trapping at or near the active channel/dielectric interfaces.

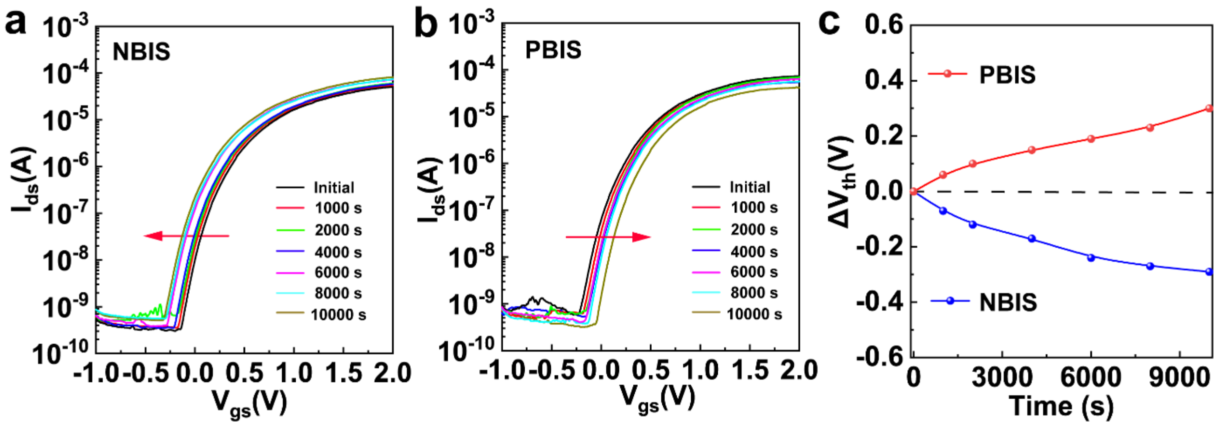

Fig. S7 Electrical stability of fully printed ITO TFTs under white light illumination. Evolution of transfer curves of the ITO TFTs as a function of (**a**) NBIS and (**b**) PBIS. (**c**) Threshold voltage shifts (ΔV_{th}) as a function of bias stress time. The NBIS/PBIS was performed in air at room temperature under white LED light illumination (3000 lux), and the applied gate bias was -/+ 1 V, respectively.

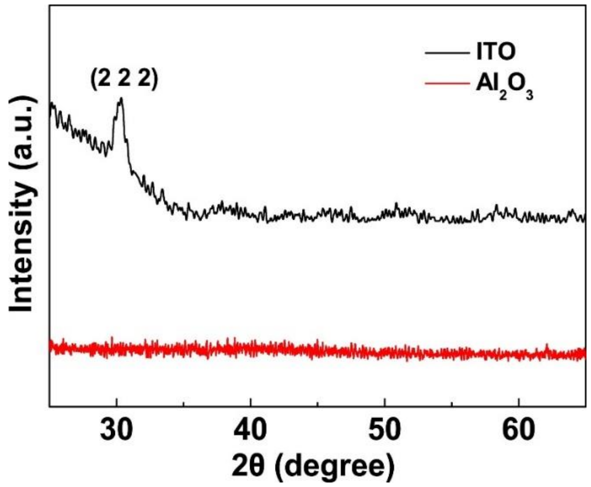

Fig. S8 X-ray diffraction patterns of printed ITO and Al_2O_3 films on SiO_2 substrates. The XRD patterns revealed the printed ITO film is polycrystalline and Al_2O_3 film is amorphous.

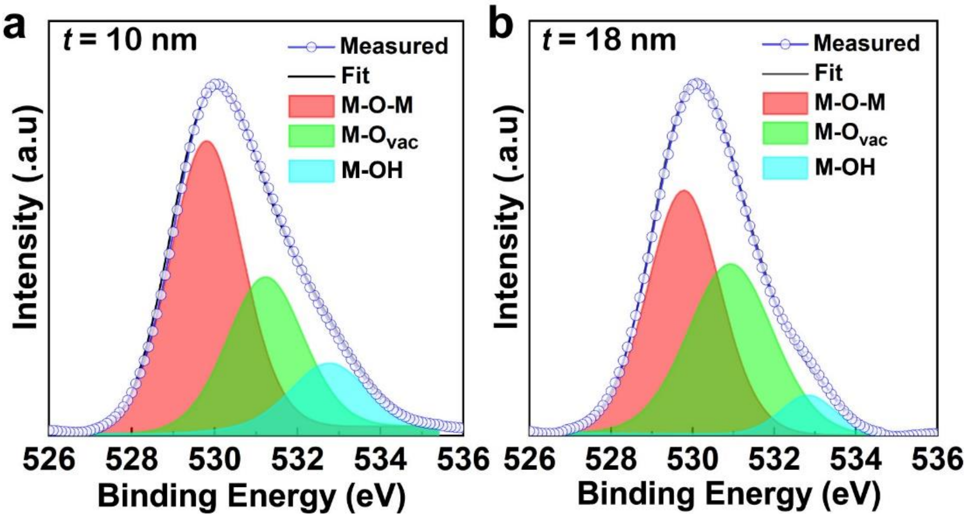

Fig. S9 XPS analysis of ITO films with different thicknesses. XPS O-1*s* spectra collected from printed (**a**) ITO channel (10 nm thick) and (**b**) ITO contacts (18 nm thick) films

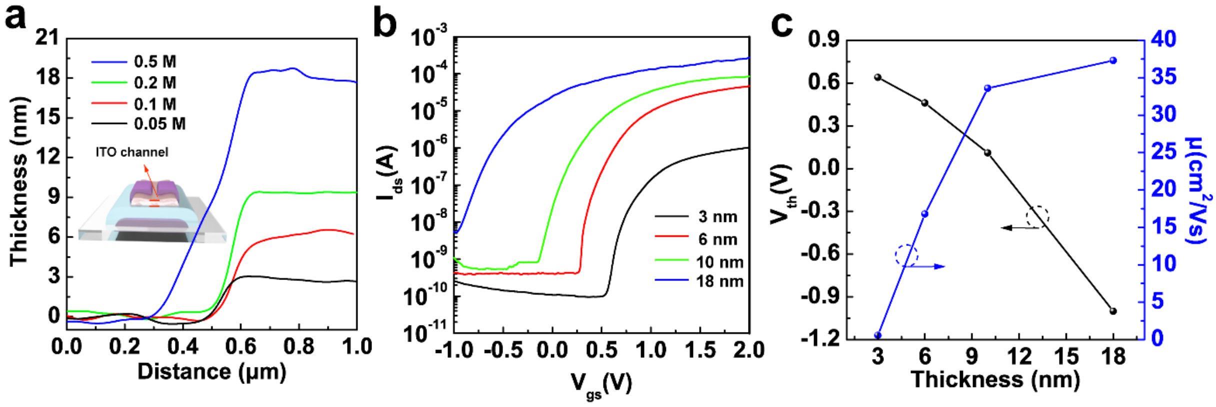

Fig. S10 The electrical characteristics of ITO TFTs with different channel thicknesses. (**a**) Cross-sectional scanning of ITO channel films by AFM. Inset illustrates the structure of the fully printed TFT. (**b**) Transfer curves of fully-printed TFTs with different channel thicknesses. (**c**) Extracted μ_{sat} and V_{th} as a function of ITO thickness

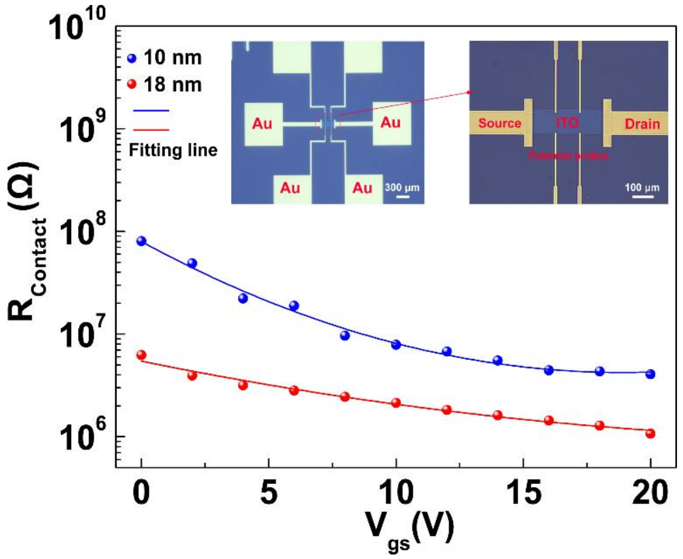

Fig. S11 Analysis of contact resistivity by gated four-probe (GFP) measurements. ITO channel was spin-coated on Si/SiO_2 substrate and patterned by photolithography and wet etching. Cr/Au (3/30 nm) electrodes were patterned and deposited via e-beam evaporation.

The contact resistance between the 10 nm, 18 nm ITO channel and the Cr/Au electrode decreases with the increase of the gate voltage. Thicker ITO has lower contact resistance. Inset illustrates optical images of the ITO GFP devices.

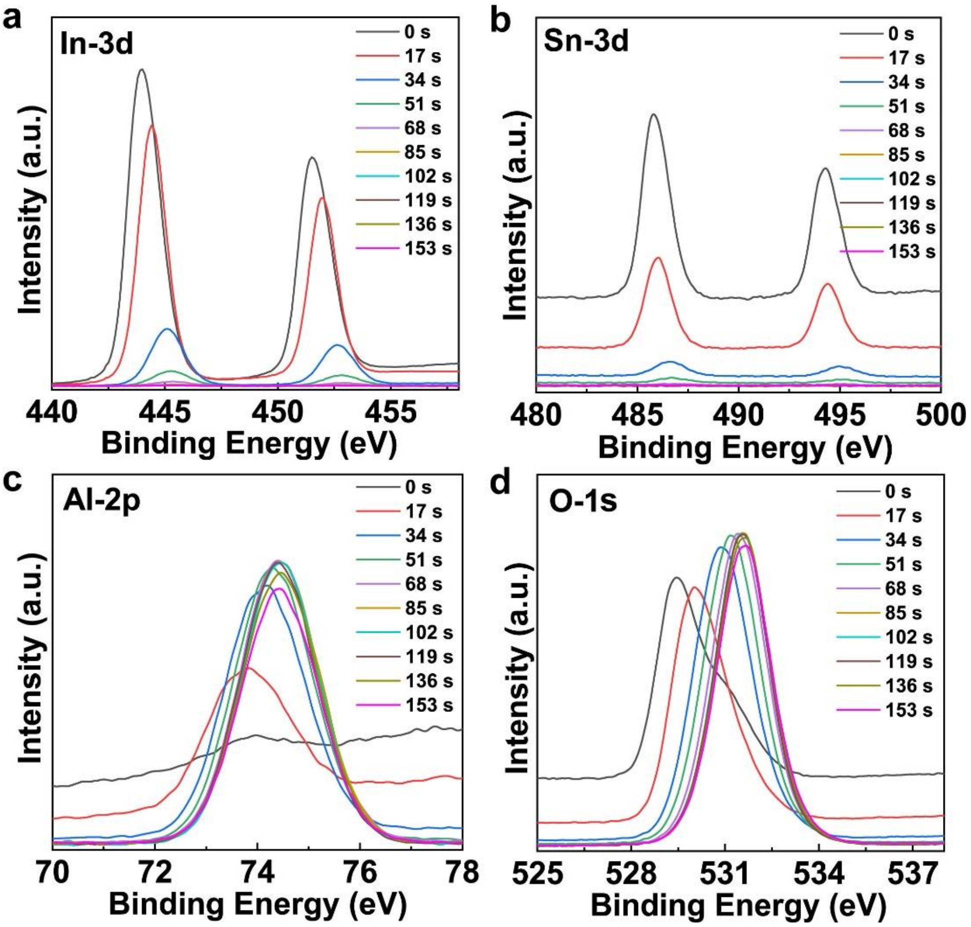

Fig. S12 XPS depth profile of the ITO/Al_2O_3 heterostructures as a function of etching time. (**a**) In-3*d*, (**b**) Sn-3*d* (**c**) Al-2*p*, and (**d**) O-1*s* core-level XPS spectra of the ITO/Al_2O_3 films, where the intensities of In-3*d* and Sn-3*d* peaks decrease with time while that of Al-2*p* shows opposite trend.

Table S1 Comparation of inverters performance based on solution-processed metal oxide TFTs

Deposition method	Channel materials	Inverter load	V_{dd} (V)	Gain (V/V)	References
	In_2O_3	Resistor	4	9.7	[S1]
	In_2O_3	Resistor	2.5	5	[S2]
	In_2O_3	Resistor	3	6	[S3]
Spin-coating	In_2O_3	Resistor	10	10.6	[S4]
	ZTO	CMOS	40	10	[S5]
	ZTO	NMOS	15	23.2	[S6]
	ZTO	Resistor	2.5	7.3	[S7]

	ZnO	CMOS	1	18	[S8]
	IZO	Resistor	4	4.46	[S9]
	IGZO	CMOS	40	50	[S10]
	IGZO	CMOS	50	5	[S11]
	IGZO	NMOS	10	1.83	[S12]
	ZnO	NMOS	15	70	[S13]
	IGZO	Resistor	5	19.8	[S14]
Inkjet-printing	In_2O_3	CMOS	1.5	18	[S15]
	In_2O_3	Resistor	4	16	[S16]
	In_2O_3	CMOS	1.5	21	[S17]
	ZnO	Resistor	2	8	[S18]
	IGZO	CMOS	10.5	12	[S19]
	In_2O_3	NMOS	20	45	[S20]
	IGZO	NMOS	0.5	2.5	[S21]
Fully-printing	IGO	Resistor	2	5	[S22]
	ITO	NMOS	3	181	**This work**

Supplementary References

[S1] C. Fan, A. Liu, Y. Meng, Z. Guo, G. Liu et al., Solution-processed SrOx-gated oxide thin-film transistors and inverters. IEEE Trans. Electron Dev. **64**, 4137 (2017). https://doi.org/10.1109/TED.2017.2742060

[S2] L. Zhu, G. He, Y. Long, B. Yang, J. Lv, Eco-Friendly, Water-induced In_2O_3 thin films for high-performance thin-film transistors and inverters. IEEE Trans. Electron Dev. **65**, 2870 (2018). https://doi.org/10.1109/TED.2018.2824336

[S3] T. Zhao, C. Zhao, J. Zhang, I.Z. Mitrovic, E.G. Lim et al., Enhancement on the performance of eco-friendly solution-processed InO/AlO thin-film transistors via lithium incorporation. J. Alloy. Compd. **829**, 154458 (2020). https://doi.org/10.1016/j.jallcom.2020.154458

[S4] Z. Guo, A. Liu, Y. Meng, C. Fan, B. Shin et al., Solution-processed ytterbium oxide dielectrics for low-voltage thin-film transistors and inverters. Ceram. Int. **43**, 15194 (2017). https://doi.org/10.1016/j.ceramint.2017.08.052

[S5] R.D. Chandra, M. Rao, K. Zhang, R.R. Prabhakar, C. Shi et al., Tuning electrical properties in amorphous zinc tin oxide thin films for solution processed electronics. ACS Appl. Mater. Interfaces **6**, 773, (2014). https://doi.org/10.1021/am401003k

[S6] S.-P. Tsai, C.-H. Chang, C.-J. Hsu, C.-C. Hu, Y.-T. Tsai et al., MESFETs and inverters based on amorphous zinc-tin-oxide thin films prepared at room temperature. ECS J. Solid State Sci. Technol. **4**, 176 (2015). https://doi.org/10.1063/1.5038941

[S7] B. Yang, G. He, Y. Zhang, C. Zhang, Y. Xia et al., Solution-processed DyOx for aging diffusion ZnSnO transistors and applications in low-voltage-operating logic circuits. IEEE Trans. Electron Dev. **66**, 3479 (2019). https://doi.org/10.1109/TED.2019.2924089

[S8] K.G. Cho, H.J. Kim, H.M. Yang, K.H. Seol, S.J. Lee et al., Sub-2 V, transfer-stamped organic/inorganic complementary inverters based on electrolyte-gated transistors. ACS Appl. Mater. Interfaces **10**, 40672 (2018). https://doi.org/10.1021/acsami.8b13140

[S9] G. He, W. Li, Z. Sun, M. Zhang, X. Chen, Potential solution-induced HfAlO dielectrics and their applications in low-voltage-operating transistors and high-gain inverters. RSC Adv. **8**, 36584 (2018). https://doi.org/10.1039/C8RA07813K

[S10] A. Liu, H. Zhu, Y.Y. Noh, Molecule charge transfer doping for p-channel solution-processed copper oxide transistors. Adv. Funct. Mater. **30**, 2002625 (2020). https://doi.org/10.1002/adfm.202002625

[S11] A. Liu, H. Zhu, W.T. Park, S.J. Kang, Y. Xu et al., Room-temperature solution-synthesized p-type copper(i) iodide semiconductors for transparent thin-film transistors and complementary electronics. Adv. Mater. 30, 1802379 (2018). https://doi.org/10.1002/adma.201802379

[S12] D. Kim, Y. Kim, K.-Y. Choi, D. Lee, H. Lee, A solution-processed operational amplifier using direct light-patterned a-InGaZnO TFTs. IEEE Trans. Electron Dev. **65**, 1796 (2018). https://doi.org/10.1109/TED.2018.2817689

[S13] F.F. Vidor, T. Meyers, K. Müller, G. I. Wirth, U. Hilleringmann, Inverter circuits on freestanding flexible substrate using ZnO nanoparticles for cost-efficient electronics. Solid-State Electron. **137**, 16 (2017). https://doi.org/10.1016/j.sse.2017.07.011

[S14] Y. Zhang, G. He, W. Wang, B. Yang, C. Zhang et al., Aqueous-solution-driven HfGdOx gate dielectrics for low-voltage-operated α-InGaZnO transistors and inverter circuits. J. Mater. Sci. Technol. **50**, 1 (2020). https://doi.org/10.1016/j.jmst.2020.03.007

[S15] T.T. Baby, S.K. Garlapati, S. Dehm, M. Häming, R. Kruk et al., A general route toward complete room temperature processing of printed and high performance oxide electronics. ACS Nano **9**, 3075 (2015). https://doi.org/10.1021/nn507326z

[S16] S.Y. Kim, K. Kim, Y.H. Hwang, J. Park, J. Jang et al., High-resolution electrohydrodynamic inkjet printing of stretchable metal oxide semiconductor transistors with high performance. Nanoscale **8**, 17113 (2016). https://doi.org/10.1039/C6NR05577J

[S17] S.K. Garlapati, T.T. Baby, S. Dehm, M. Hammad et al., Ink-jet printed CMOS electronics from oxide semiconductors. Small **11**, 3591 (2015). https://doi.org/10.1002/smll.201403288

[S18] K. Hong, S.H. Kim, K.H. Lee, C.D. Frisbie, Printed, sub-2V ZnO electrolyte gated transistors and inverters on plastic. Adv. Mater. **25**, 3413 (2013). https://doi.org/10.1002/adma.201300211

[S19] C. Chen, Q. Yang, G. Chen, H. Chen, T. Guo, Solution-processed oxide complementary inverter via laser annealing and inkjet printing. IEEE Trans. Electron Devices **66**, 4888 (2019). https://doi.org/10.1109/TED.2019.2941264

[S20] J. Leppaniemi, K. Eiroma, H.S. Majumdar, A. Alastalo, In_2O_3 thin-film transistors via inkjet printing for depletion-load nMOS inverters. IEEE Electron Device Lett. **37**, 445 (2016). https://doi.org/10.1109/LED.2016.2529183

[S21] B.K. Sharma, A. Stoesser, S.K. Mondal, S.K. Garlapati, M.H. Fawey et al., High-performance all-printed amorphous oxide FETs and logics with electronically compatible electrode/channel interface. ACS Appl. Mater. Interfaces **10**, 22408 (2018). https://doi.org/10.1021/acsami.8b04892

[S22] Y. Li, L. Lan, S. Hu, P. Gao, X. Dai et al., Fully printed top-gate metal–oxide thin-film transistors based on scandium-zirconium-oxide dielectric. IEEE Trans. Electron Devices **66**, 445 (2019). https://doi.org/10.1109/TED.2018.2877979
